# Are Separated Fathers Less or More Involved in Childrearing than Partnered Fathers?

**DOI:** 10.1007/s10680-021-09593-1

**Published:** 2021-10-20

**Authors:** Tara Koster, Teresa Castro-Martín

**Affiliations:** 1 Department of Sociology, Utrecht University, Padualaan 14, 3584 CH Utrecht, The Netherlands; 2 Institute of Economy, Geography and Demography, Spanish National Research Council (CSIC), Madrid, Spain

**Keywords:** Separation, Father involvement, Partnered fathers, Resident fathers, Shared residence fathers, Nonresident fathers, Post-separation residence arrangements

## Abstract

Separated fathers are generally assumed to be less involved with their children than partnered fathers. Yet, extant research on separated fathers has mainly focused on nonresident fathers without taking into consideration the existing diversity in post-separation residence arrangements. In fact, separated resident and shared residence fathers may possibly be more involved than partnered fathers, because the former likely bear primary childcare responsibilities, while the latter often act as secondary caregivers. This study extends previous research by investigating father involvement via regular care and leisure activities across a full range of separated fathers, and how it compares to that of partnered fathers, as well as whether patterns differ by father’s education. Data from the New Families in the Netherlands survey (*N* = 1592) reveal that as compared to partnered fathers, shared residence fathers and especially resident fathers are more actively involved in the regular care of their child, whereas nonresident fathers are less involved. Results are similar for leisure, except that partnered fathers are similarly involved as shared residence fathers in this activity. Education also matters: involvement of fathers across different post-separation residence arrangements is more similar to that of partnered fathers when being highly educated. These findings suggest that including resident and shared residence fathers in the picture offers a more optimistic view of fathers’ post-separation parenting role, because these separated fathers are actually more actively involved in childrearing than partnered fathers.

**Supplementary Information:**

The online version contains supplementary material available at 10.1007/s10680-021-09593-1.

## Introduction

Over the last decades, there has been a remarkable shift in the social and cultural norms shaping fathers’ involvement with their children, reflecting changes in family gender roles and the division of care and paid work. Alongside their breadwinning responsibilities, fathers are nowadays expected to assume a nurturing role and to get involved in their children’s direct care (Hook & Wolfe, 2012). In response to the evolving definition of fatherhood and the increased recognition of the important role fathers play on child development and well-being, the literature on fathering has become more extensive and varied, although the bulk of research still focuses on parenting behaviors of partnered fathers^1^ compared to partnered mothers’ (Lamb, 2000).

In this general context of increasing father involvement in children’s upbringing, the rise in union dissolution poses an important challenge to the continuity and quality of father–child relationships (Härkönen, 2014). In the recent past, mother sole custody was the norm, and the degree of involvement of separated fathers^2^ was assessed by the frequency of father–child contact, economic support, and participation in childrearing decisions, but less so by fathers’ engagement in childcare (Seltzer, 1991). However, besides the cultural shift in the normative expectations of fathers’ nurturing roles, the continuing involvement of separated fathers in their children’s lives has been reinforced by social policies oriented to promote more gender-equal engagement in childrearing in all types of families (Pilkauskas & Schneider, 2020) and legislative changes toward more gender-neutral parental custody laws (Lamb, 2000; McIntosh, 2009). Hence, the social and cultural shifts toward more involved fatherhood have altered living and care arrangements after separation. Post-separation residence arrangements have diversified over the last decades, with a small rise of resident fathers (i.e., children residing primarily with their father) and a steep rise of fathers in shared residence (i.e., children residing alternately with each parent) (Bernardi & Mortelmans, 2021). Ample visitation rights for nonresident fathers have also increased their involvement in parenting activities (Waller et al., 2018).

In this changing demographic, social, and cultural context, the literature on separated fathers has evolved from the initial focus on child support payments and father–child contact to a growing emphasis on fathers’ caregiving. However, existing studies on separated fathers have mainly focused on nonresident fathers, and less attention has been devoted to other types of residence arrangements (but see Bastaits & Mortelmans, 2017; Bastaits et al., 2014; Hook & Chalasani, 2008) which were once uncommon, but are now on the rise. The literature consistently shows that separated fathers are less involved with their children than partnered fathers (e.g., Carlson et al., 2017; Grätz, 2017), yet this generalization does not take into consideration the existing diversity in post-separation residence arrangements. In fact, separated resident and shared residence fathers might possibly be more involved in childrearing than partnered fathers, because the former place great emphasis on their parenting role and the latter often act as secondary caregivers.

The present study contributes to the existing literature, first, by taking into account the existing variety of separated fathers’ residential contexts. By focusing on a full range of post-separation residence arrangements (i.e., resident father, shared residence father, nonresident father), we examine father involvement of all types of separated fathers, and how they compare to partnered fathers. Second, we test whether differences in father involvement across residence arrangements vary by father’s education. Educational attainment is consistently found to be a key factor in explaining the level and type of parental engagement (Monna & Gauthier, 2008; Sullivan, 2010). Prior research has shown that high-educated partnered fathers tend to be more involved with their children than the lower educated, because the former are more likely to adopt modern fatherhood norms and often have the resources (i.e., time and money) that make involvement easier (Köppen et al., 2018; Sayer et al., 2004). High-educated nonresident fathers have also been found to be more involved in childrearing (Cheadle et al., 2010; Kalmijn, 2015). Less is known about how education affects fathers’ active parenting in other post-separation residence arrangements, and this study tries to fill this gap.

We use data from the New Families in the Netherlands (NFN) survey (Poortman et al., 2014, 2018). The strength of the NFN is that it includes extensive information about father involvement in a broad range of parent–child activities (i.e., regular care and leisure) of large samples of both married/cohabiting fathers and divorced/separated fathers. Additionally, the sample of divorced/separated fathers includes a relatively large number of resident and shared residence fathers, which allows a comprehensive examination of father involvement across a full variety of residential contexts.

## Background and Hypotheses

Father involvement is a multidimensional and continually evolving construct, which encompasses a wide range of behavioral, cognitive, and affective practices. Notwithstanding the growing literature on fathering (Marsiglio et al., 2000), there is still no consensual theoretical framework to guide research on father involvement, possibly because it constitutes a moving target, as social expectations of paternal roles are continuously evolving over time. The pioneering conceptualization of father involvement by Lamb et al. (1987) identified three key dimensions: accessibility (physical and psychological availability to the child), engagement (direct interactions with the child through caretaking and shared activities), and responsibility (organizing and managing child’s care and welfare). Subsequent studies have expanded the concept of father involvement to include aspects such as warmth, responsiveness, emotional support, or supervision (Pleck, 2010), and have fostered multidisciplinary approaches (Cabrera & Tamis-LeMonda, 2013). Despite the wide plurality in conceptualizations and measurements of paternal involvement across studies, disciplines, and societies, there is nonetheless a common shift in focus from quantity to quality of time spent with children and an increasing attention to diversity in fathers’ parenting across different family contexts (Schoppe-Sullivan & Fagan, 2020).

### Residential Status and Father Involvement

Partnered and separated fathers have different opportunities and constraints with regard to how they fulfill their parental role and dedicate time and effort to childcare. In the literature, we find three arguments to expect different levels of father involvement according to residential status. A first argument focuses on the structural position of being a primary caregiver. Men and women have been long socialized into different gender roles, with women having the greatest responsibility for childrearing, whereas men were expected to assume the primary breadwinner role (Craig & Mullan, 2011). In the last decades, the so-called new fatherhood has emerged, shifting the focus of fatherhood from its breadwinner role to its nurturing role (Hook & Wolfe, 2012). Nonetheless, although time use studies have documented that the amount of time partnered fathers spend with their children has increased, partnered mothers continue to bear major responsibility for care tasks (Craig & Mullan, 2011). Many partnered fathers are still “part-timers” in childrearing, performing a complementary role rather than being their child’s primary caregiver (Craig, 2006). After separation, when there is a reduction of care provided by the mother, resident and shared residence fathers take on the role of primary caregiver, irrespective of whether they are repartnered (Bastaits & Mortelmans, 2017). Taking on nontraditional roles and responsibilities to provide the child’s daily care—even if this only applies half of the time in the case of shared residence—they will generally be more involved than partnered fathers (Hilton & Devall, 1998; Spruijt & Duindam, 2010). Nonresident fathers are not in a primary caregiver position, just like most partnered fathers. Although there has been a notable increase in the frequency of nonresident father–child contact over divorce cohorts (Westphal et al., 2014), living in separate households poses major challenges for active fathering. Nonresident fathers are more constrained in their access to their child than partnered fathers, because the former often face practical barriers (e.g., geographical distance, time and visitation rights) which likely limit their level and forms of parenting involvement (Hawkins et al., 2006).

A second argument focuses on mothers’ gatekeeping behavior. Although some studies suggest that partnered mothers generally value and actively promote their partner’s involvement with their child (i.e., positive gatekeeping) (Puhlman & Pasley, 2013; Walker & McGraw, 2000), others have shown that partnered mothers’ gatekeeping can also be restrictive for fathers (i.e., negative gatekeeping) (Allen & Hawkins, 1999; Gaunt, 2008). Partnered mothers may inhibit fathers’ active engagement in parenting so they can retain primary responsibility for childrearing (Fagan & Barnett, 2003), or because they do think of fathers providing a lower standard of care than they themselves deliver (Bianchi & Milkie, 2010). Negative gatekeeping may be even more pronounced in a post-separation context for the nonresident father, because of a protective resident mother. Although not all resident mothers may “gatekeep” the father’s access to the child (Sano et al., 2008), they often play a major role in deciding how much time the father spends with the child by limiting (face-to-face) father–child contact to either the formal court prescriptions or the agreed-upon informal visitation arrangements (Pruett et al., 2006). Nonresident fathers may, thus, be more hindered from being actively involved with their child than partnered fathers. By contrast, (shared) resident fathers are not likely to experience negative gatekeeping by the mother. For resident fathers, negative gatekeeping would be ineffective as nonresident mothers are not in the position of primary caregiver. For fathers in shared residence, negative gatekeeping would be impractical because this particular living arrangement requires extensive cooperation between the parents (Nielsen, 2011).

Third, research has shown that separated parents generally have more feelings of guilt toward their children than partnered parents, as the former may feel guilty about the separation and its potential negative consequences for the child (Kalmijn, 2018). For (shared) resident fathers, feelings of guilt may positively affect their involvement. Because these fathers co-reside with their child at least half of the time and act as primary caregiver, they may respond to these feelings of guilt by trying to compensate for the potentially harmful consequences of separation, or for the reduction of care provided by the mother. (Shared) resident fathers may be particularly motivated to show that they are a “good” parent by dedicating extra time and effort to their child’s needs. Nonresident fathers are often more constrained to be engaged in their child’s daily life, but they might try to compensate their guilt feelings by focusing on shared leisure activities with their child—sometimes described as “Disneyland dads” (Stewart, 1999).

Prior research has consistently shown that nonresident fathers are less involved with their child than partnered fathers (Carlson et al., 2017; Hawkins et al., 2006). The few studies that have compared father involvement between partnered fathers and separated fathers in the less common residence arrangements indicate that resident fathers are more involved than partnered fathers (Bastaits & Mortelmans, 2017; Hilton & Devall, 1998; Hook & Chalasani, 2008). Shared residence fathers have been found to display similar levels of father involvement as partnered fathers (Bastaits & Mortelmans, 2017). Note that mentioned studies include different measures of father involvement (e.g., paternal control, emotional support, parent–child activities) and different samples (e.g., parenting reported by parents or children, recent or older data collection), which may make it difficult to compare the findings, and that relatively small sample sizes of separated fathers in infrequent residential arrangements often preclude firm conclusions. Building on prior research and based on the arguments developed above, we expect that:

#### H1a

Shared residence fathers and especially resident fathers are more involved with their child than partnered fathers.

#### H1b

Nonresident fathers are less involved with their child than partnered fathers.

### Residential Status, Father’s Education, and Father Involvement

Highly educated fathers are generally well aware of the positive impact of father involvement on children’s development and well-being. Well-educated fathers are also more likely to embrace modern fatherhood norms and to have more financial resources to invest in their children (Kalmijn, 2015; Monna & Gauthier, 2008). Hence, high-educated fathers are typically more involved in childrearing than low-educated fathers—yet the extent may depend on the residential context.

Previous studies have shown that partnered fathers—who generally perform a secondary role in caregiving—take on more of the responsibility for parenting tasks when being highly educated (Köppen et al., 2018; Sayer et al., 2004). As compared to partnered fathers, educational attainment may positively influence father involvement to a lesser extent for resident and shared residence fathers because of a ceiling effect. Regardless of their educational level, (shared) resident fathers bear major responsibility for childrearing in their primary caregiver position. High-educated (shared) resident fathers may be somewhat more involved with their child than their low-educated counterparts, yet it is unlikely that we find large differences across educational strata. Differences in parenting involvement between partnered fathers and (shared) resident fathers may thus be smaller among the highly educated because of greater involvement of high-educated partnered fathers.

The literature on nonresident fathers has also documented that the well-educated are more engaged in parenting than the lower educated (Manning et al., 2003; Westphal et al., 2014). It is plausible that educational attainment positively affects parenting involvement of nonresident fathers to a greater extent than that of partnered fathers. High-educated nonresident fathers may be more motivated than high-educated partnered fathers to take on an active parenting role to compensate for the fact that they do not live with their child and the child’s potential loss of social capital, for instance by increasing the quantity and quality of father–child interactions (Cheadle et al., 2010). The financial aspect may also be relevant for nonresident fathers (Kalmijn, 2015). Well-educated fathers tend to have more economic resources that facilitate contact, especially if the child lives at some distance (Cheadle et al., 2010). High-educated nonresident fathers may also be more able to contribute financially to the child’s care, thereby gaining the ex-partner’s cooperation (Coley & Hernandez, 2006). The ex-partner may allow increased visitation between the nonresident father and the child, which positively affects the father’s opportunities to be involved. These arguments suggest that differences in parenting involvement between partnered fathers and nonresident fathers may be smaller among the highly educated because of higher involvement of high-educated nonresident fathers. On the basis of these considerations, we hypothesize that:

#### H2a

Among the highly educated, the gap in father involvement between partnered fathers and (shared) resident fathers is smaller because of greater involvement of high-educated partnered fathers.

#### H2b

Among the highly educated, the gap in father involvement between partnered fathers and nonresident fathers is smaller because of greater involvement of high-educated nonresident fathers.

### The Dutch Context and Selection Issues

Since the 1970s, a new ideal of fatherhood has emerged, emphasizing fathers’ nurturing role in both partnered and post-separation families (Lamb, 2000). Many Western countries started to encourage partnered and separated fathers’ parenting involvement with new legislation and social policies ever since. In the Netherlands, partnered fathers’ engagement in childcare has been promoted to some extent, yet many policies still consider fathers as primary breadwinners and mothers as primary caregivers (Korpi et al., 2013). Since 2001 fathers are entitled to two days of paternity leave,^3^ whereas mothers get sixteen weeks of maternity leave (Plantenga & Remery, 2009). Because paternity leave is limited and there is no heavily subsidized public childcare, a “one-and-a-half earner” model has become dominant (Visser, 2002)—with the majority of two-parent families with children composed of a full-time working father and a part-time working mother who takes most responsibility for childcare. Although there has been a trend toward increasing participation of partnered fathers in childcare over the last decades, partnered mothers continue to devote almost twice as much time on their children’s care (Portegijs et al., 2018).

Regarding post-separation families, automatic continuation of joint legal custody after union dissolution—implying shared decision-making on child-related matters—is regulated by Dutch law since the late 1990s. Moreover, since 2009 the law promotes joint physical custody after union dissolution. With this emphasis on promoting continued coparenting after separation, shared residence has become an increasingly common living arrangement in the Netherlands. About one-fourth of divorced and separated parents are in a shared residence arrangement (Spruijt & Kormos, 2014). As shared residence is only encouraged rather than prescribed by Dutch law, there is often self-selection into this residence arrangement. Fathers who opt for shared residence often have a high socioeconomic status, experience little pre-separation conflict and few personal problems (Poortman & Van Gaalen, 2017). These factors likely also affect their level of post-separation parenting involvement (Cancian et al., 2014; Gunnoe & Braver, 2001). Father residence after separation is relatively uncommon, about 7% (Spruijt & Kormos, 2014). Father residence is more likely when the child is older (Poortman & Van Gaalen, 2017) and parent characteristics may also play a role (e.g., father’s high pre-separation involvement, mother’s personal problems). Selection into sole father custody may also affect post-separation fathering (Kitterød & Lyngstad, 2014). Furthermore, it is important to be aware of fathers’ possible selection into separation. Partnered fathers who experience high levels of marital conflict or who are less involved in childrearing are more likely to separate (Kalmijn, 1999). To address potential selection bias, analyses control for a wide range of parental demographic and (pre-separation) relationship characteristics.

## Data and Methods

### Data

We used the survey NFN (Poortman & Van Gaalen, 2019a, 2019b; Poortman et al., 2014, 2018). Because questions about involvement in parent–child activities were only included in Wave 2 (2015/16), the bulk of the analysis is based on Wave 2, although some information of Wave 1 (2012/13) was also used. Based on population registers, Statistics Netherlands (CBS) draw two random samples for the first wave: one among married or cohabiting heterosexual parents with minor children (i.e., partnered sample) and one among formerly married or cohabiting heterosexual parents with minor children who dissolved their union in 2010 (i.e., separated sample). For both samples, both (ex-)partners were approached by post and invited to complete an online survey. The final reminder included a written questionnaire. For the partnered sample, the response rate in Wave 1 was 45% among persons and 56% among households, yielding 2173 married or cohabiting parents. For the separated sample, the response rate was 39% on the individual level and 58% on the former household level, totaling 4481 divorced or separated parents. These response rates are comparable to other Dutch family surveys, and relatively high considering that NFN uses an online mode, but also targets a group of recently separated parents (Poortman et al., 2014).

Parents who agreed to be re-contacted for follow-up research were invited to participate in Wave 2, in which a similar procedure was followed. For the partnered sample, the retention rate—the overall percentage of participants in Wave 1 who also participated in Wave 2—was 61% among persons and 67% among households, totaling 1336 participants. For the separated sample, the retention rate amounted to 57% on the individual level and 63% on the former household level, yielding 2544 participants. An additional random sample of formerly married or cohabiting heterosexual parents with minor children who dissolved their union in 2010 was approached to participate in Wave 2 to compensate for panel attrition. The response rate was 32% among persons and 52% among former households, resulting in 920 participants in this refreshment sample. The total sample of Wave 2 contains 4800 parents, of which 1336 married/cohabiting parents and 3464 divorced/separated parents.

Regarding the partnered sample, for both waves, men were moderately underrepresented, yet selectivity on several characteristics was similar for men and women: non-western immigrants and people on low incomes were underrepresented. Additionally, the well-educated were more likely to respond in Wave 2. Regarding the separated sample, similar to Wave 1, men were moderately underrepresented, and selectivity on several criteria was similar for men and women: former cohabiters, younger people, non-western immigrants, and people on low incomes were underrepresented. Note also that having a high education and paid work were most predictive of participating again in Wave 2.

Parents provided information on a focal child who was selected in Wave 1. If at least one of the children was ten or older at the time of Wave 1, parents reported on the youngest child of ten or older. If all children were younger than ten, parents reported on the oldest child. In Wave 2 parents answered questions about the same child. In the refreshment sample, the cutoff age for selecting the focal child was thirteen years old because Wave 2 took place about three years after Wave 1.

Because the focus of this study is on father involvement, we only selected fathers (*n* = 1971). We excluded fathers who had children with a same-sex (former) partner (*n* = 4). Fathers were also excluded when the focal child was older than 18 years of age at the time of Wave 2 (*n* = 277). Fathers with another living arrangement than partnered, resident, shared residence or nonresident father were omitted (*n* = 47). We further excluded cases with missing values on the variables of interest (*n* = 51). Missing values were low, ranging from 0 to 1.2%. The final analytical sample consisted of 1592 fathers (partnered sample: *n* = 482; separated sample: *n* = 1110).

### Dependent Variables

Drawing on the key components of fathering—availability, engagement, and responsibility—posited by Lamb et al. (1987), the present study largely focuses on the engagement dimension, that is, the extent to which fathers experience shared interactions with their child. We distinguish two types of activities fathers may engage in with their children: *regular care* and *leisure*. The distinction between care and play is relevant, because partnered and nonresident fathers have been found to often enjoy the more pleasurable aspects of childcare by spending more time on leisure activities with their child (Craig, 2006; Stewart, 1999), whereas (shared) resident fathers are expected to be involved in a wide range of parent–child activities.

The measure of *regular care* is derived from five items on how often (1 = *not* to 7 = *few times per day*) fathers spent time with their child during the last month in the following activities: “having dinner together,” “helping with school or homework,” “talking with child about issues in child’s life,” “dropping child off or picking child up from school or sports,” and “doing household tasks together.” We calculated the mean score for these items combined (Cronbach’s *α* = 0.84).

The measure of *leisure* is derived from three items on how often (1 = *not* to 7 = *few times per day*) fathers spent time with their child during the last month in the following activities: “watching television,” “playing a game or doing crafts,” and “leisure activities away from home, such as to the zoo.” A scale was created by calculating the mean score (Cronbach’s *α* = 0.80). The correlation between regular care and leisure was statistically significant and positive (*r* = 0.78).^4^

### Independent Variables

*Father’s residential status.* Fathers of the partnered sample were assigned to the group of partnered fathers. Fathers of the separated sample indicated with whom the focal child lived most of the time at the time of the survey, with response categories: “with me,” “with ex-partner,” “with both parents about equally,” and “other arrangement.” We excluded fathers in the “other arrangement” category. Dummy variables were constructed for the four different groups of fathers: *partnered father* (reference group), *resident father*, *nonresident father,* and *shared residence father*.

*Father’s education*. In Wave 1 fathers reported their highest attained educational level (1 = *primary school not finished* to 10 = *post graduate*). Because the effect of education may not be linear, we generated three dummy variables: *low education* (lower secondary education or less) as the reference group, *medium education* (upper secondary education and vocational training) and *high education* (tertiary education).

### Control Variables

As mentioned earlier, we controlled for factors that may be related to selection into separation as well as into pre-separation residence arrangements, namely pre-separation father involvement, pre-separation level of conflict and pre-separation union type—for partnered fathers those variables refer to their current partnership. We also controlled for factors that the literature has documented to be associated with father involvement: parents’ age, mother’s education, father’s work hours, child’s gender and age, and number of children^5^ (Carlson et al., 2017; Cheadle et al., 2010; Grätz, 2017; Landale & Oropesa, 2001; Manning et al., 2003; Sayer et al., 2004).

To measure *(pre-separation) involvement*, partnered fathers reported at Wave 1 who did most (1 = *I much more often than partner* to 5 = *partner much more often than me*) of five care tasks (e.g., putting child to bed) during their current relationship. For separated fathers, the items and response categories were similar, yet they reported on who did most during the relationship with their ex-partner. We recoded the items in the direction of the father’s contribution, so that a higher score indicated that his involvement was higher than his (ex-)partner’s. The mean score was computed (Cronbach’s *α* = 0.84). For (*pre-separation) conflict*, fathers indicated how often five different conflict situations (e.g., fierce arguments) occurred during the past year (partnered sample) or in the final year before separation (separated sample). Answers ranged from 1 (= *never*) to 4 (= *often*). A scale was created by taking the mean (Cronbach’s α = 0.89). *(Pre-separation) union type* is a dummy for whether the father’s relationship with the (ex-)partner was 0 “*cohabitation*” or 1 “*marriage / registered partnership.*” A registered partnership is a form of legal cohabitation offering almost the same rights as marriage (6% in the sample). *Parents’ age* is measured in years. We constructed variables for the father’s and the mother’s age, based on father’s reports. *Mother’s education* was reported by the father, and was measured in the same way as father’s education. *Father’s work hours* refer to the number of contractual hours that fathers worked per week. If they did not have a paid job at the time of the survey, they were assigned zero hours. Work hours of over 60 were recoded to 60. Because of small regression coefficients, we divided father’s work hours by 10. *Child’s gender* is a dummy for whether the focal child was a 0 “*boy”* or 1 “*girl”*. *Child’s age* is the focal child’s age measured in years. *Number of children* includes the number of children fathers had or adopted with their (ex-)partner. Table 1 shows descriptive statistics of all variables used in the analyses across fathers’ residential contexts.

**Table 1:** Range, mean and standard deviation of the variables in the analyses *Source:* New Families in the Netherlands, Wave 1, 2

		Partnered fathers		Resident fathers		Shared residence fathers		Nonresident fathers	
	Range	*M*	*SD*	*M*	*SD*	*M*	*SD*	*M*	*SD*
Regular care	1–7	4.30	0.89	4.76	0.89	4.55	0.84	3.08	1.29
Leisure	1–7	4.05	1.09	4.31	1.20	4.20	1.03	3.11	1.35
Father’s education									
Low education	0–1	0.16	^a^	0.17	^a^	0.10	^a^	0.22	^a^
Medium education	0–1	0.34	^a^	0.40	^a^	0.30	^a^	0.37	^a^
High education	0–1	0.50	^a^	0.43	^a^	0.59	^a^	0.41	^a^
*Controls*									
(Pre-separation) involvement	1–5	2.46	0.63	3.13	0.80	2.90	0.59	2.75	0.63
(Pre-separation) conflict	1–4	1.50	0.40	2.33	0.76	2.08	0.71	2.32	0.76
(Pre-separation) union type									
Cohabitation	0–1	0.25	^a^	0.17	^a^	0.26	^a^	0.23	^a^
Marriage	0–1	0.75	^a^	0.83	^a^	0.74	^a^	0.77	^a^
Father’s age	28–73	47.59	6.35	48.18	6.79	47.06	6.06	46.71	6.82
Mother’s age	26–73	45.17	5.78	45.23	5.79	44.22	5.65	44.10	6.08
Mother’s education									
Low education	0–1	0.17	^a^	0.41	^a^	0.20	^a^	0.36	^a^
Medium education	0–1	0.35	^a^	0.35	^a^	0.33	^a^	0.37	^a^
High education	0–1	0.49	^a^	0.23	^a^	0.47	^a^	0.27	^a^
Father’s work hours (× 10)	0–6	3.64	1.09	3.24	1.46	3.55	1.20	3.48	1.38
Child’s gender									
Boy	0–1	0.47	^a^	0.54	^a^	0.52	^a^	0.51	^a^
Girl	0–1	0.53	^a^	0.46	^a^	0.48	^a^	0.49	^a^
Child’s age	2–18	12.67	3.37	14.31	3.09	12.79	2.99	12.78	3.29
Number of children	1–8	2.14	0.85	2.05	0.85	1.92	0.72	1.89	0.82
*N* of respondents		482		111		426		573	

^a^Standard deviation (SD) not presented for discrete variables

### Analytical Strategy

We, first, describe and compare the characteristics of partnered, resident, shared residence, and nonresident fathers in Table 1. We also added Table 2 with more descriptive information only concerning separated fathers (e.g., post-separation repartnering), which may be relevant for parenting involvement, but cannot be included in the multivariate analysis because it encompasses both partnered and separated fathers. Next, linear regression analyses were performed, estimating three models for both regular care and leisure (Table 3). Model 1 includes only father’s residential status to assess whether there are observed differences in father involvement by residence arrangements. This model should be interpreted with caution because it does not control for possible selection factors. In Model 2, we added father’s education and the controls, to examine the net impact of residential status on father involvement. Model 3 includes interactions between residential status and father’s education to test whether differentials in father involvement by residential status vary across educational strata. Because of few cases in some groups (e.g., resident fathers with low education: *n* = 19), we dichotomized the education variable: 0 “less than tertiary education” (i.e., low and medium education) and 1 “tertiary education.” Wald tests were conducted to test for interactions with the three dummies for residential status simultaneously.

## Results

### Profile of Fathers by Residential Status

Table 1 shows that the mean amount of father involvement in regular care and leisure across residence arrangements differed in expected ways: it was highest among resident fathers, followed by shared residence fathers, partnered fathers, and nonresident fathers, respectively. Whereas differences between partnered and (shared) resident fathers were not that large, nonresident fathers clearly lagged behind in their parenting involvement. As regards education, shared residence fathers stood out with the highest proportion of highly educated—in line with prior research (Poortman & Van Gaalen, 2017)—and nonresident fathers with the highest proportion of lower educated. Mother’s education is also particularly low in the case of resident fathers. Somewhat unexpectedly, (pre-separation) involvement was higher among all types of separated fathers than partnered fathers, but differences between separated fathers were as anticipated: shared residence fathers, and especially resident fathers, were more involved prior to the separation than nonresident fathers. In line with prior research (Kalmijn, 1999), there was a clear distinction in (pre-separation) conflict between partnered fathers and separated fathers, with the former experiencing less conflict. Pre-separation conflict also varied among separated fathers: resident and nonresident fathers reported similar levels of pre-separation conflict, whereas fathers in shared residence reported lower levels.

Table 2 provides more detailed information on separated fathers. Whereas the majority of shared residence fathers (66%) and nonresident fathers (70%) had repartnered—be it co-residing with a new partner or in a LAT relationship—resident fathers were less often in a new partnership (45%). Among repartnered fathers, only a relatively small proportion had joint children with the new partner (6%, 12% and 15% among resident, shared residence, and nonresident fathers, respectively). Co-residing with a new partner who had children from a prior union, and thus having stepchildren, was more common, particularly among nonresident fathers (25%). The mean amount of monthly parent–child contact shows that the residence groups differed in expected ways (i.e., resident fathers: 24 days; shared residence fathers: 14 days; nonresident fathers: 5 days). Although there was a nontrivial proportion of nonresident fathers who had no face-to-face contact with their child at all (9%), the large majority saw their child once per month or more often (84%). Lastly, we observe that for most separated fathers, travel time to the ex-partner’s house was low (i.e., 15 min or less), yet this was more so the case for fathers in shared residence (80%) than for resident fathers (59%) and nonresident fathers (58%)—which corroborates findings from previous research (Thomas et al., 2018).

**Table 2:** Range, mean and standard deviation of additional variables for separated fathers *Source:* New Families in the Netherlands, Wave 2

		Resident fathers		Shared residence fathers		Nonresident fathers	
	Range	*M*	*SD*	*M*	*SD*	*M*	*SD*
Repartnering							
No partner	0–1	0.56	^a^	0.35	^a^	0.30	^a^
LAT partner	0–1	0.22	^a^	0.30	^a^	0.18	^a^
Co-residing partner	0–1	0.23	^a^	0.36	^a^	0.52	^a^
Stepchildren							
No stepchildren	0–1	0.68	^a^	0.61	^a^	0.63	^a^
LAT and stepchildren	0–1	0.18	^a^	0.24	^a^	0.12	^a^
Co-residing and stepchildren	0–1	0.14	^a^	0.16	^a^	0.25	^a^
Joint children with new partner							
No joint children	0–1	0.94	^a^	0.88	^a^	0.85	^a^
Joint children	0–1	0.06	^a^	0.12	^a^	0.15	^a^
Monthly father–child contact	0–28	23.92	3.67	13.57	1.91	4.63	3.31
Yearly face-to-face contact with child							
Never	0–1					0.09	^a^
1–11 times per year	0–1					0.07	^a^
Once per month or more often	0–1					0.84	^a^
Travel time to ex-partner’s house							
15 min or less	0–1	0.59	^a^	0.80	^a^	0.58	^a^
Between 16–59 min	0–1	0.30	^a^	0.16	^a^	0.30	^a^
60 min or more	0–1	0.11	^a^	0.03	^a^	0.12	^a^

^a^Standard deviation (SD) not presented for discrete variables

### Differences in Father Involvement across Residential Contexts

Model 1 in Table 3 only includes father’s residential status. Consistent with the descriptive results, resident and shared residence fathers were more involved with their child in both regular care and leisure activities than partnered fathers (reference category), and nonresident fathers were less involved. All coefficients were statistically significant.

**Table 3:** Regression analyses for variables predicting father involvement *Source:* New Families in the Netherlands, Wave 1, 2

	Regular care				Leisure		
	Model 1	Model 2	Model 3		Model 1	Model 2	Model 3
Father's residential status (ref. = partnered father)							
Resident father	0.46**^ab^ (.11)	0.59**^ab^ (.11)	0.66**^ab^ (.14)		0.26*^b^ (.12)	0.43**^ab^ (.12)	0.59**^ab^ (.16)
Shared residence father	0.25**^c^ (.07)	0.18*^c^ (.07)	0.24*^c^ (.10)		0.15*^c^ (.08)	0.11^c^ (.08)	0.18^c^ (.11)
Nonresident father	−1.22** (.06)	−1.17** (.07)	−1.32** (.09)		−0.94** (.07)	−0.92** (.08)	−1.04** (.10)
Father’s education (ref. = low education)							
Medium education		0.09^d^ (.07)				0.14 ~ (.08)	
High education		0.25** (.08)				0.17* (.09)	
Father’s education (ref. = less than tertiary education)			0.10 (.09)				0.02 (.10)
*Interactions of father’s education with:*							
Resident father			−0.16 (.21)				−0.36 (.23)
Shared residence father			−0.09 (.13)				−0.11 (.15)
Nonresident father			0.35** (.12)				0.28* (.14)
*Controls*							
(Pre-separation) involvement		0.21** (.04)	0.22** (.04)			0.15** (.04)	0.16** (.04)
(Pre-separation) conflict		−0.09* (.04)	−0.09* (.04)			−0.10* (.04)	−0.10* (.04)
(Pre-separation) union type		−0.03 (.06)	−0.02 (.06)			−0.01 (.07)	−0.01 (.07)
Father’s age		0.00 (.01)	0.00 (.01)			0.00 (.01)	0.00 (.01)
Mother’s age		0.00 (.01)	0.00 (.01)			−0.01 (.01)	−0.01 (.01)
Mother’s education (ref. = low education)							
Medium education		0.07 (.07)	0.08 (.06)			0.02 (.07)	0.04 (.07)
High education		0.14* (.07)	0.15* (.07)			0.01 (.08)	0.03 (.08)
Father’s work hours (× 10)		0.04 ~ (.02)	0.04* (.02)			0.04 (.02)	0.04 ~ (.02)
Child’s gender (ref. = boy)		−0.04 (.05)	−0.04 (.05)			−0.14* (.05)	−0.13* (.05)
Child’s age		−0.09** (.01)	−0.09** (.01)			−0.12** (.01)	−0.12** (.01)
Number of children		−0.11** (.03)	−0.12** (.03)			−0.14** (.04)	−0.14** (.04)
*Adjusted R*^*2*^	0.292	0.389	0.394		0.151	0.285	0.289
*N* of respondents	1592	1592	1592		1592	1592	1592

^a^The difference between resident father and shared residence father is significant (two-sided *p* < .05). For regular care Model 1, this difference is only marginally significant (two-sided *p* < .10)

^b^The difference between resident father and nonresident father is significant (two-sided *p* < .01)

^c^The difference between shared residence father and nonresident father is significant (two-sided *p* < .01)

^d^The difference between father's medium education and father's high education is significant (two-sided *p* < .01)

~ Two-sided *p* < .10; *Two-sided *p* < .05; **Two-sided *p* < .01

Model 2 takes into account father’s education and the control variables. Starting with the results for regular care, resident fathers (*b* = 0.59) and shared residence fathers (*b* = 0.18) were more involved with their child than partnered fathers. Father involvement was lower for nonresident fathers as compared to partnered fathers (*b* = −1.17). Additional analyses with shared residence fathers as reference category furthermore showed that resident fathers were more involved in regular care than shared residence fathers (*b* = 0.41). As regards leisure activities, resident fathers showed greater levels of involvement than partnered fathers (*b* = 0.43), whereas nonresident fathers showed lower levels of involvement than partnered fathers (*b* = −0.92). The difference in father involvement between shared residence fathers and partnered fathers, however, was not statistically significant, indicating that these fathers were similarly involved in leisure activities. Effect sizes were generally modest to large. Only two small effect sizes were found: for regular care, the difference between partnered and shared residence fathers (0.15 = 0.18/SD(Y) with SD(Y) = 1.24) and, for leisure, the difference between resident and shared residence fathers (0.25 = 0.32/1.28). These findings generally confirm H1a and H1b that compared to partnered fathers, shared residence fathers and especially resident fathers were more involved with their child, whereas nonresident fathers were less involved. Only with regard to leisure activities, we did not find that shared residence fathers were more involved than partnered fathers.

Comparing the coefficients between Models 1 and 2, we observe that all differentials in parenting involvement by residential status remain statistically significant, except for the case of shared residence fathers, which no longer differed from partnered fathers in their engagement in leisure activities after controls. We also observe an increase in the coefficient of sole residence and a small reduction of the coefficient of shared residence and nonresidence on the level of father involvement in both regular care and leisure, after including control variables. These findings indicate that it is necessary to control for possible selection into separation and post-separation residence arrangements in order to avoid under- or overestimation of the differentials in father involvement by residential status.

Model 2 further shows the association between father’s education and father’s involvement. Fathers with a high level of education were more involved in both regular care and leisure than fathers with a low education (regular care: *b* = 0.25; leisure: *b* = 0.17). Additional analyses showed that high-educated fathers were also more involved in regular care than fathers with a medium education (*b* = 0.16).

The rest of the covariates controlled for in Model 2 show, in general, the expected sign. (Pre-separation) involvement was positively related to father involvement for both regular care and leisure. Fathers with a high-educated (ex-)partner were more involved in regular care than fathers with a low-educated (ex-)partner. (Pre-separation) conflict, the child’s higher age, and having a larger number of children were associated with lower father involvement in both regular care and leisure. Fathers were less involved in leisure activities if the child was a girl.

### Differences in Father Involvement across Residential Contexts by Education

Model 3 in Table 3 includes the interactions between father’s residential status and father’s education. For both regular care and leisure, Wald tests showed that adding interactions improved model fit (regular care: χ^*2*^(3) = 5.45; *p* = 0.001; leisure: χ^*2*^(3) = 4.22; *p* = 0.006). The statistically significant and positive interaction effects with nonresident father (regular care: *b* = 0.35; leisure: *b* = 0.28) indicated that the gap in involvement between nonresident fathers and partnered fathers narrowed among the highly educated.

**Fig. 1:**
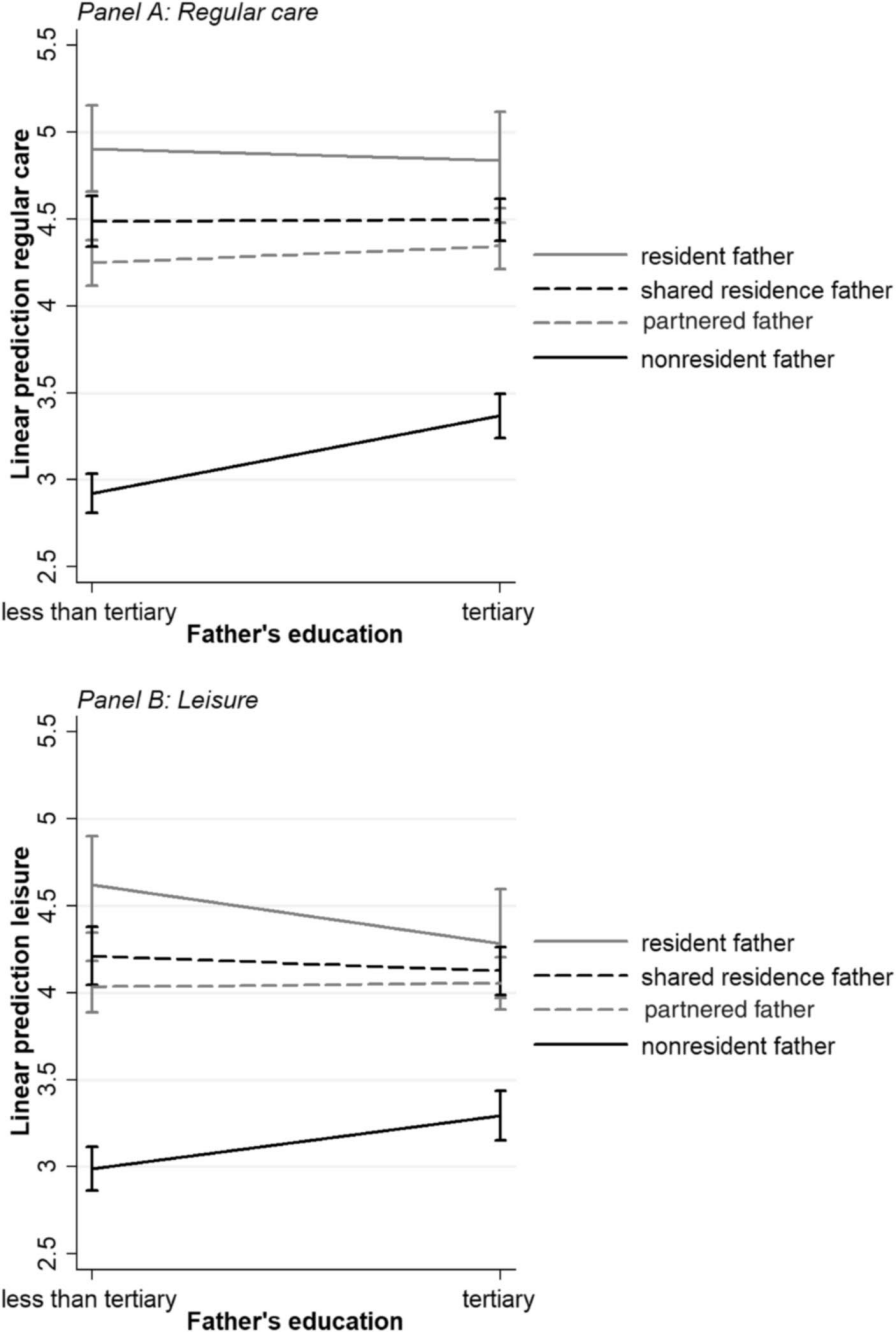
Father involvement in regular care and leisure by father’s residential status and education

To better interpret the interaction model, we graphically represented the results in Fig. 1. This figure shows predicted father involvement in regular care (Panel A) and leisure (Panel B) and the 95% confidence intervals for different combinations of father’s education (at values 0 “less than tertiary education” and 1 “tertiary education) and residence arrangements. For regular care, Fig. 1 (Panel A) shows the expected differences in father involvement across residential contexts when fathers had no tertiary education. Partnered fathers with tertiary education were only slightly more involved in regular care than their partnered counterparts with a lower education. Shared residence fathers were equally involved and resident fathers slightly less involved when they had tertiary education. As a result, when fathers were highly educated, there was no statistically significant gap in involvement between partnered and shared residence fathers, and the gap somewhat narrowed between partnered and resident fathers. Nonresident fathers’ involvement increased to a greater extent than that of partnered fathers at a higher educational level. Although the difference in father involvement between partnered and nonresident fathers remained statistically significant among the highly educated, the gap in father involvement was smaller.

For leisure, Fig. 1 (Panel B) shows that among fathers without tertiary education, resident fathers were more involved and nonresident fathers less involved than partnered fathers. Shared residence fathers were similarly involved as partnered fathers. The influence of father’s education across residence arrangements followed roughly similar patterns as for regular care. A tertiary education was related to greater involvement for nonresident fathers, but only slightly for partnered fathers. Resident fathers, and shared residence fathers only to a limited extent, were less involved in leisure when being highly educated. As a result, when being highly educated, partnered, shared residence, and resident fathers were similarly involved in leisure activities, whereas nonresident fathers lagged behind—although the gap was clearly smaller.

Overall, these findings confirm H2b that among the highly educated, the gap in father involvement between partnered fathers and nonresident fathers was smaller because of greater involvement of high-educated nonresident fathers. We found weak support for H2a that among the highly educated, the gap in father involvement between partnered fathers and (shared) resident fathers was smaller because of greater involvement of high-educated partnered fathers. High-educated partnered fathers were only slightly more involved, whereas high-educated (shared) resident fathers were equally involved or less involved—particularly in leisure—than their less educated counterparts. The gap in father involvement between partnered fathers and (shared) resident fathers did narrow among the highly educated, but not for the reasons that we had anticipated. A possible explanation for the lower involvement of high-educated resident fathers might be that only a few resident fathers were low-educated in our sample (*n* = 19), and these fathers are likely to be selective (e.g., mother’s personal problems), which may have driven their higher level of involvement.

### Robustness Checks

To test the robustness of our findings, we first ran propensity score analysis (PSM). Including possible selection factors as controls in multivariate regression models has been systematically done in previous studies to address potential selection issues, but PSM provides an alternative method to minimize selection bias on observed variables—although bias from unobserved factors may still remain (McCaffrey et al., 2013). We re-estimated the regression models with the PSM matched sample of separated and partnered fathers. We first adjusted the distribution of demographic and socioeconomic characteristics of the four groups of fathers (i.e., partnered, resident, shared residence, and nonresident fathers), to make the groups balanced on the characteristics that may affect both group assignment and father involvement. The method we used was propensity score weighting for multiple treatments using generalized boosted models (McCaffrey et al., 2013). The covariates we included for the propensity score matching were: father’s education, age and work hours, mother’s age and education, child’s gender and age, number of children, and (pre-separation) union type. (Pre-separation) conflict and involvement were excluded because including these two variables not only resulted in a sample size (*N* = 1095) that differed substantially from the actual sample size (*N* = 1592), but also imbalance between the groups of fathers remained in these two variables. After the propensity score matching, the weighted sample (*N* = 1346) consisted of 402 partnered fathers, 60 resident fathers, 377 shared residence fathers, and 507 nonresident fathers who were balanced on the abovementioned variables. In a next step, we performed propensity-weighted regressions estimating father involvement on the weighted sample. In the weighted regression models, we only controlled for (pre-separation) conflict and involvement, as these variables were not included in the propensity score matching (McCaffrey et al., 2013). The findings of these analyses can be consulted in Table S1 in the supplementary materials, and showed similar patterns as the ones presented in the paper.

Second, the literature often distinguishes regular care activities in practical and developmental dimensions of care (Kendig & Bianchi, 2008). We rerun separate models for practical care activities (i.e., dropping child off or picking child up from school or sports; having dinner together; doing household tasks together) and for positive engagement activities that are likely to foster child development (i.e., helping with school or homework; talking about issues in child’s life). Results (in Table S2) showed that differentials in father involvement by residential status were similar for both types of care activities.

Third, in our sample 9% of nonresident fathers did not see their child in the past year (*n* = 52). To check that differences in father involvement between nonresident fathers and fathers in the other residence arrangements were not driven by this group of parents who had no contact at all with their child, we performed additional analyses excluding these non-involved nonresident fathers. These analyses, shown in Table S3, yielded similar results.

Fourth, because the main analyses included partnered fathers, we could not control for factors that were only relevant for separated fathers, such as new family responsibilities. In additional analyses confined to the separated sample, we examined the influence of new partnerships (in Table S4, Model 2a) and additional (step/joint) children (in Table S4, Model 2b) on separated fathers’ involvement across residential contexts.^6^ For nonresident fathers, we found that it was having additional children rather than repartnering that affected their level of involvement: those who co-resided with a new partner who had children from a prior union (i.e., stepchildren) were less involved in regular care and leisure—although effect sizes were small (regular care: 0.22 = 0.29/1.29; leisure: 0.24 = 0.33/1.35). Shared residence fathers’ involvement remained unchanged, which suggests that the higher engagement of shared residence fathers in childcare is not significantly affected by their new family responsibilities.

## Discussion

Both partnered and separated fathers are increasingly willing and socially expected to be actively involved in childrearing. At the same time, post-separation residence arrangements have become increasingly heterogeneous, particularly due to the rise in shared residence. Research on separated fathers, however, has primarily focused on nonresident fathers, and has consistently found that they are less involved than partnered fathers. This general impression of less involved separated fathers might be misleading and needs to be nuanced, taking into consideration the changing context of post-separation care arrangements. Expanding on previous studies, we examined father involvement in regular care and leisure activities across a full range of post-separation residence arrangements and how it compares to that of partnered fathers, and whether patterns differed by father’s education.

Although separation certainly triggers disruptions in parenting and a reconfiguration of family roles (Härkönen, 2014), this study’s analysis of recent Dutch data has shown that separated fathers were either more or less involved than partnered fathers, depending on their post-separation residence arrangements. In line with findings from previous research (Carlson et al., 2017; Hawkins et al., 2006), nonresident fathers were less involved with their child than partnered fathers. Nonresident fathers are typically more constrained in their access to their child than partnered fathers. Limited visitation schedules or high levels of negative gatekeeping behavior by the mother may hinder their involvement. Note that for a substantial number of nonresident fathers in our sample, travel time to the ex-partner’s house was low, indicating that their lower involvement may also be a matter of choice: nonresident fathers may disengage themselves from the parental role and feel less obligated to be involved in childrearing (Haux & Platt, 2021). In contrast, shared residence fathers, and particularly resident fathers, showed higher levels of involvement in regular care activities than partnered fathers. Although both partnered fathers and (shared) resident fathers co-reside with the child, it seems that the latter take on more of the responsibility for parenting tasks. These fathers may also be highly motivated to perform the “good parent” role to compensate for the potentially adverse consequences of the separation. Nonetheless, for leisure activities, we did not find that shared residence fathers were more involved than partnered fathers. This finding is not surprising as previous studies have shown that partnered fathers generally enjoy the more pleasurable aspects of childcare and are more reluctant to engage in day-to-day practical care (Craig, 2006; Stewart, 1999).

The current study also found that when fathers were highly educated, involvement of fathers in all post-separation residence arrangements was more similar to that of partnered fathers. Possibly driven by modern fatherhood norms and resources, and in line with findings from previous studies (Sayer et al., 2004; Westphal et al., 2014), highly educated partnered and nonresident fathers were found to be more involved with their child than their lower educated counterparts—although this was clearly stronger for nonresident fathers. This latter finding could indicate that highly educated nonresident fathers choose to often visit the child, which corroborate findings from previous studies showing that well-educated nonresident fathers nowadays have more contact with their children than in the past (Westphal et al., 2014). High levels of visitation allow for more active fathering (Waller et al., 2018). For both resident and shared residence fathers, a high education affected their level of fathering involvement to a lesser extent than that of partnered and nonresident fathers. Because (shared) resident fathers already bear primary responsibility for the child’s day-to-day care, this may be why there are no large observed differences in their level of involvement by educational attainment. This conclusion needs to be nuanced for resident fathers, who were found to be less engaged in regular care and leisure when being highly educated, but, as noted, this result might be related to the small number of low-educated resident fathers in the sample.

The study also has some limitations that deserve consideration. First, although we have tried to minimize selection bias by controlling for pre-separation factors and performing propensity score analysis, self-selection into separation and post-separation residence may still underlie some of the differences found in father involvement across residence arrangements, so solid causal claims cannot be made. Second, less than 1% of fathers in our sample reported on a child under the age of four. Differences in father involvement between partnered fathers and (shared) resident fathers may be particularly large when children are very young, as partnered fathers may experience maternal gatekeeping (Gaunt, 2008), whereas (shared) resident fathers may be more responsive to younger children’s greater need of childcare. Third, fathers with low income and low education were underrepresented in both the separated and partnered sample. Less involved fathers might also be underrepresented as they might have lower interest to fill in a survey on family relationships. The underrepresentation of less involved fathers and those with low socioeconomic status, which are more likely to be in a nonresidence than a shared/sole residence arrangement, might lead to an overestimation of the childcare contribution of participating nonresident fathers. Hence, differences in father involvement may in fact be larger than our results suggest. Fourth, fathers in all residence arrangements may have overreported their level of involvement as fathers tend to overestimate their engagement with children (Pasley & Braver, 2004) or feel embarrassed of their actual involvement. Future research may use time diaries to minimize social desirability bias.

Notwithstanding these limitations, the current study provides valuable insights on active fathering across residential contexts. Despite new legislation and social policies to encourage fathers’ post-separation involvement (McIntosh, 2009; Pilkauskas & Schneider, 2020), thus far empirical evidence on actual father involvement in the case of shared and father residence remains scarce. As one of the first to examine father involvement across a variety of residential contexts, this study showed that including resident and shared residence fathers offers a more optimistic view of fathers’ post-separation parenting role, because these fathers are actually more involved in childrearing than partnered fathers. In fact, for some of them the reconfiguration of family roles triggered by the separation may have increased their level of engagement with their children. Education also mattered, as the involvement of fathers in the different post-separation residence arrangements was more similar to that of partnered fathers when they were highly educated.

## Supplementary Information

Below is the link to the electronic supplementary material.

Supplementary file1 (DOCX 28 kb)

## Footnotes

1 In this study we use the term “partnered fathers” to refer broadly to fathers who are married or in a cohabiting union with the child’s mother.

2 In this study we use the term “separated fathers” to refer broadly to fathers who dissolved their marriage or cohabiting union with the child’s mother.

3 Paternity leave has been recently extended (in 2019), but only to five days.

4 Fathers could also fill out “not applicable” (e.g., child is too old or too young) for all items involved in the two dependent variables. Fathers were treated as having a missing value on these particular items if they did so. They were included in the scale when having a non-missing value on at least one of the items. For three items we found missing values of about 10%, as these items indicate activities for which children are too young (helping with school; dropping off/picking up) or too old (playing game/doing crafts).

5 Wave 1 information was used for some controls as this information was no longer asked in Wave 2 (i.e., partnered sample: mother’s education; separated sample: mother’s education, pre-separation involvement, conflict, and union type, and number of children).

6 Resident fathers could not be included in this analysis because of small number of cases (e.g., resident fathers co-residing with new partner: *n* = 25; resident fathers with joint children: *n* = 7).

## Declarations

### Data Availability

The datasets analyzed in the current study are archived in the Dans-KNAW repository (Wave 1: https://doi.org/10.17026/dans-xj8-83k7; Wave 2: https://doi.org/10.17026/dans-24y-n8s4) and only available on request.
