# Supplementary material for: Are Separated Fathers Less or More Involved in Childrearing than Partnered Fathers?
Source: Eur J Popul. 2021 Oct 20;37(4-5):933–57. doi: 10.1007/s10680-021-09593-1 (PMC8575742; doi:10.1007/s10680-021-09593-1)
Supplement: Supplementary file 1 — Supplementary file1 (DOCX 28 kb) [file 10680_2021_9593_MOESM1_ESM.docx]

**Supplementary materials**

**Table S1** Propensity weighted regression analyses for variables predicting father involvement

|  | Regular care | | Leisure | |
| --- | --- | --- | --- | --- |
|  | Model 2 | Model 3 | Model 2 | Model 3 |
| Father’s residential status (ref. = partnered father) |  |  |  |  |
| Resident father | 0.52**^ab^  (.13) | 0.61**^ab^  (.19) | 0.40*^ab^  (.18) | 0.58*^ab^  (.25) |
| Shared residence father | 0.19**^c^  (.07) | 0.24**^c^  (.09) | 0.13^c^  (.09) | 0.20^c^  (.12) |
| Nonresident father | -1.18**  (.08) | -1.37**  (.10) | -0.91**  (.09) | -1.08**  (.13) |
| Father’s education (ref. = less than tertiary education) |  | 0.12  (.08) |  | -0.04  (.11) |
|  |  |  |  |  |
| Interactions of father’s education with: |  |  |  |  |
| * resident father |  | -0.18  (.24) |  | -0.36  (.31) |
| * shared residence father |  | -0.11  (.12) |  | -0.13  (.15) |
| * nonresident father |  | 0.40**  (.14) |  | 0.38*  (.16) |
| *Controls* |  |  |  |  |
| (Pre-separation) involvement | 0.24**  (.05) | 0.25**  (.05) | 0.17**  (.06) | 0.19**  (.06) |
| (Pre-separation) conflict | -0.05  (.05) | -0.05  (.05) | -0.08  (.06) | -0.09  (.06) |
|  |  |  |  |  |
| *R^2^* | 0.326 | 0.340 | 0.171 | 0.182 |

*Note:* ^a^ The difference between resident father and shared residence father is significant (two-sided *p* <.05). For leisure Models 2 and 3 this difference is only marginally significant (two-sided *p* <.10). ^b^ The difference between resident father and nonresident father is significant (two-sided *p* <.01). ^c^ The difference between shared residence father and nonresident father is significant (two-sided *p* <.01).

~ Two-sided *p* < .10; * Two-sided *p* < .05; ** Two-sided *p* < .01. *Source:* New Families in the Netherlands, Wave 1, 2.

**Table S2** Regression analyses for variables predicting father involvement in practical and developmental care activities

|  | Practical care | | Developmental care | |
| --- | --- | --- | --- | --- |
|  | Model 2 | Model 3 | Model 2 | Model 3 |
| Father’s residential status (ref. = partnered father) |  |  |  |  |
| Resident father | 0.58**^ab^  (.14) | 0.62**^ab^  (.18) | 0.60*^ab^  (.12) | 0.67**^ab^  (.15) |
| Shared residence father | 0.19*^c^  (.09) | 0.20^c^  (.12) | 0.15*^c^  (.08) | 0.24*^c^  (.11) |
| Nonresident father | -1.04**  (.08) | -1.21**  (.11) | -1.28**  (.07) | -1.43**  (.10) |
| Father’s education (ref. = low education) |  |  |  |  |
| Medium education | 0.09^d^  (.09) |  | 0.08^d^  (.08) |  |
| High education | 0.31**  (.09) |  | 0.20*  (.08) |  |
| Father’s education (ref. = less than tertiary education) |  | 0.12  (.11) |  | 0.07  (.10) |
|  |  |  |  |  |
| Interactions of father’s education with: |  |  |  |  |
| * resident father |  | -0.12  (.25) |  | -0.18  (.22) |
| * shared residence father |  | 0.01  (.16) |  | -0.14  (.14) |
| * nonresident father |  | 0.37*  (.15) |  | 0.35**  (.13) |
|  |  |  |  |  |
| *Adjusted R^2^* | 0.286 | 0.289 | 0.368 | 0.373 |
| *N* of respondents | 1588 | 1588 | 1591 | 1591 |

*Note*: Analyses control for (pre-separation) involvement, (pre-separation) conflict, (pre-separation) union type, father’s and mother’s age, mother’s education, father’s work hours, child’s gender and age, and number of children. ^a^ The difference between resident father and shared residence father is significant (two-sided *p* <.05). ^b^ The difference between resident father and nonresident father is significant (two-sided *p* <.01). ^c^ The difference between shared residence father and nonresident father is significant (two-sided *p* <.01). ^d^ The difference between father's medium education and father's high education is significant (two-sided *p* <.01). For developmental care this difference is only marginally significant (two-sided *p* <.10). ~ Two-sided *p* < .10; * Two-sided *p* < .05; ** Two-sided *p* < .01. *Source:* New Families in the Netherlands, Wave 1, 2.

**Table S3** Regression analyses for variables predicting father involvement, excluding nonresident fathers who did not see their child in the past year

|  | Regular care | | Leisure | |
| --- | --- | --- | --- | --- |
|  | Model 2 | Model 3 | Model 2 | Model 3 |
| Father’s residential status (ref. = partnered father) |  |  |  |  |
| Resident father | 0.50**^ab^  (.10) | 0.55**^ab^  (.14) | 0.34*^ab^  (.12) | 0.48**^ab^  (.16) |
| Shared residence father | 0.13~^c^  (.07) | 0.18~^c^  (.09) | 0.05^c^  (.08) | 0.12^c^  (.11) |
| Nonresident father | -1.03**  (.07) | -1.18**  (.09) | -0.78**  (.07) | -0.90**  (.10) |
| Father’s education (ref. = low education) |  |  |  |  |
| Medium education | 0.12^d^  (.07) |  | 0.16*  (.08) |  |
| High education | 0.26**  (.07) |  | 0.17*  (.08) |  |
| Father’s education (ref. = less than tertiary education) |  | 0.09  (.09) |  | 0.02  (.10) |
|  |  |  |  |  |
| Interactions of father’s education with: |  |  |  |  |
| * resident father |  | -0.14  (.19) |  | -0.33  (.22) |
| * shared residence father |  | -0.07  (.12) |  | -0.09  (.14) |
| * nonresident father |  | 0.34**  (.12) |  | 0.26~  (.13) |
|  |  |  |  |  |
| *Adjusted R^2^* | 0.365 | 0.369 | 0.263 | 0.265 |
| *N* of respondents | 1540 | 1540 | 1540 | 1540 |

*Note*: Analyses control for (pre-separation) involvement, (pre-separation) conflict, (pre-separation) union type, father’s and mother’s age, mother’s education, father’s work hours, child’s gender and age, and number of children. ^a^ The difference between resident father and shared residence father is significant (two-sided *p* <.05). ^b^ The difference between resident father and nonresident father is significant (two-sided *p* <.01). ^c^ The difference between shared residence father and nonresident father is significant (two-sided *p* <.01). ^d^ The difference between father's medium education and father's high education is significant (two-sided *p* <.05). ~ Two-sided *p* < .10; * Two-sided *p* < .05; ** Two-sided *p* < .01. *Source:* New Families in the Netherlands, Wave 1, 2.

**Table S4** Regression analyses for variables predicting father involvement of shared residence and nonresident fathers, including new family responsibilities

|  | Shared residence fathers | | | | Nonresident fathers | | | |
| --- | --- | --- | --- | --- | --- | --- | --- | --- |
|  | Regular care | | Leisure | | Regular care | | Leisure | |
|  | Model 2a | Model 2b | Model 2a | Model 2b | Model 2a | Model 2b | Model 2a | Model 2b |
| Repartnering (ref. = no partner) |  |  |  |  |  |  |  |  |
| LAT partner | 0.13  (.09) |  | -0.01  (.11) |  | -0.18  (.15) |  | -0.20  (.16) |  |
| Co-residing partner | 0.12  (.10) |  | -0.02  (.12) |  | -0.16  (.12) |  | -0.20  (.13) |  |
| Stepchildren (ref. = no stepchildren) |  |  |  |  |  |  |  |  |
| LAT and stepchildren |  | 0.10  (.09) |  | 0.02  (.11) |  | 0.03^a^  (.16) |  | 0.00 ^a^  (.17) |
| Co-residing and stepchildren |  | 0.01  (.11) |  | -0.05  (.13) |  | -0.29*  (.12) |  | -0.33**  (.12) |
| Joint children with new partner (ref. = no joint children) |  | -0.07  (.13) |  | 0.06  (.16) |  | 0.05  (.15) |  | 0.00  (.16) |
| Father’s education (ref. = low education) |  |  |  |  |  |  |  |  |
| Medium education | -0.16^b^  (.14) | -0.19^b^  (.14) | -0.22  (.17) | -0.22  (.17) | 0.24~^b^  (.14) | 0.24~^b^  (.14) | 0.30*  (.15) | 0.30*  (.15) |
| High education | -0.01  (.14) | -0.03  (.14) | -0.22  (.17) | -0.21  (.17) | 0.53**  (.15) | 0.50**  (.15) | 0.42**  (.16) | 0.38*  (.16) |
|  |  |  |  |  |  |  |  |  |
| *Adjusted R^2^* | 0.175 | 0.172 | 0.184 | 0.183 | 0.109 | 0.114 | 0.147 | 0.153 |
| *N* of respondents | 425 | 426 | 425 | 426 | 573 | 573 | 573 | 573 |

*Note*: Analyses control for pre-separation involvement, pre-separation conflict, pre-separation union type, father’s and mother’s age, mother’s education, father’s work hours, child’s gender and age, and number of children. ^a^ The difference between co-residing partner and stepchildren, and LAT partner and stepchildren is significant (two-sided *p<*.05). For leisure this difference is only marginally significant (two-sided *p* <.10). ^b^ The difference between father's medium education and father's high education is significant (two-sided *p* <.05). For regular care this difference is only marginally significant (two-sided *p* <.10). ~ Two-sided *p* < .10; * Two-sided *p* < .05; ** Two-sided *p* < .01. *Source:* New Families in the Netherlands, Wave 1, 2.
